# Wheat ear counting using K-means clustering segmentation and convolutional neural network

**DOI:** 10.1186/s13007-020-00648-8

**Published:** 2020-08-06

**Authors:** Xin Xu, Haiyang Li, Fei Yin, Lei Xi, Hongbo Qiao, Zhaowu Ma, Shuaijie Shen, Binchao Jiang, Xinming Ma

**Affiliations:** 1 Henan Agricultural University, Zhengzhou, 450002 China; 2 Henan Grain Crops Collaborative Innovation Center, Zhengzhou, 450002 China

**Keywords:** Wheat ear counting, Crop yield, Deep learning, CNN, K-means, Segmentation, Recognitions

## Abstract

**Background:**

Wheat yield is influenced by the number of ears per unit area, and manual counting has traditionally been used to estimate wheat yield. To realize rapid and accurate wheat ear counting, K-means clustering was used for the automatic segmentation of wheat ear images captured by hand-held devices. The segmented data set was constructed by creating four categories of image labels: non-wheat ear, one wheat ear, two wheat ears, and three wheat ears, which was then was sent into the convolution neural network (CNN) model for training and testing to reduce the complexity of the model.

**Results:**

The recognition accuracy of non-wheat, one wheat, two wheat ears, and three wheat ears were 99.8, 97.5, 98.07, and 98.5%, respectively. The model *R*^2^ reached 0.96, the root mean square error (RMSE) was 10.84 ears, the macro F1-score and micro F1-score both achieved 98.47%, and the best performance was observed during late grain-filling stage (*R*^2^ = 0.99, RMSE = 3.24 ears). The model could also be applied to the UAV platform (*R*^2^ = 0.97, RMSE = 9.47 ears).

**Conclusions:**

The classification of segmented images as opposed to target recognition not only reduces the workload of manual annotation but also improves significantly the efficiency and accuracy of wheat ear counting, thus meeting the requirements of wheat yield estimation in the field environment.

## Background

Wheat is one of the most important food crops that play a significant role in national food security. The wheat grain-filling period is the key growth period that determines yield formation, and the number of ears per unit area is an important factor of yield [1–3]. Thus, it is of great significance to estimate wheat yield by rapidly determining the ear number. During production, the manual counting method is often used to estimate production, which is time-consuming and labor-intensive. Conversely, machine vision, machine learning, and image processing technologies can be used to rapidly and accurately identify wheat ear per unit area. This is of great significance to wheat yield estimation and provides technical support and a foundation for the acquisition of wheat plant phenotypic information.

The development of high spatial resolution computer vision-based phenotype identification [4–6] has produced high-throughput phenotyping platforms [7]. Image processing technology has been used to identify the number of ears of wheat [8, 9], but the methods focus on texture features, color segmentation, morphological extraction, and other feature extraction methods. Cointault et al. [10] used a color texture image analysis method based on mixed space to realize the recognition and counting of wheat ear. Fernandez-Gallego et al. [11] used local maximum peak values to count ears based on RGB color images in field conditions [12]. The current recognition methods based on image processing technology require extensive artificial image feature extraction, which places high demand on the environment and technology.

In recent years, machine learning has been shown to have a significant advantage in the field of machine vision, such as in image segmentation and object recognition [13–15]. Zhu et al.[16] used a support vector machine segmentation (SVM) model to realize wheat ear counting, and Li et al. [17] used a neural network based on texture features to detect ears, the accuracy of which exceeded 80%. Hasan et al. [18] used an in-depth learning method to detect and count wheat ears, achieving a highest accuracy of 94%. Madec et al. [19] used CNN to identify wheat ears from low-spatial-resolution RGB images. Machine learning methods provide automatic feature extraction and excellent parameter adjustment, which greatly reduce manual feature extraction and interpretation. However, the use of machine learning to identify grains requires the manual extraction of the image feature building the data set. Thus, these methods are prone to some human error and also have the disadvantages of identification inaccuracy caused by the adhesion of multiple wheat ears. At the same time, a simple and rapid counting system for wheat ears is lacking, and the development of such a system would have a significant impact on wheat production.

Image processing methods are influenced by the extraction of image features, lighting conditions, shadows, and complex backgrounds [20], and the requirements of the environment and technology are limited by the data set itself [21]. Although wheat ear recognition methods based on CNN are advantageous, image features (wheat ears) need to be manually extracted in order to construct the dataset [22]. To overcome the above issues, we propose the use of image processing technology to extract wheat ear features rapidly, combining this with CNN to reduce the workload of manual labeling and improve the recognition accuracy.

In this paper, we use mobile devices to rapidly acquire wheat ear images in the field environment and extract the contour features of the wheat ears automatically based on the K-means clustering algorithm, thus reducing the workload of the manual extraction of wheat ear features. On this basis, we constructed an image classification dataset with four types of labels: non-wheat ear, one wheat ear, two wheat ears, and three wheat ears. Ultimately, a CNN model was constructed to realize the rapid and accurate identification of wheat ears in the complex field environment as well as to provide technical support for the accurate yield estimation of wheat.

## Materials and methods

### Field experiments

Experiment 1 was conducted in Xuchang, China, at the Campus of Henan Agricultural University in 2018, 2019 in the experimental farm (34°08′N, 113°48′E). The Xuchang site is in the center of China, with a typical temperate and monsoonal climate. The previous crop was soybean. The tested wheat varieties included AK58, XN509, YM49, and ZM27. The experimental plot was 10 m long, 2 m wide, and with a row spacing of 20 cm. A split-plot design was adopted and was repeated three times. In order to facilitate sampling and field operation, a 1 m wide channel was set up between each plot. Nitrogen fertilization was applied as ammonium nitrate in the winter at rates of 120 kg ha^−1^ for every year, and watering once in overwintering period and jointing period respectively.

Experiment 2 was conducted in Yuanyang, Xinxiang, China, at the Yuanyang Science and Education Park (35°6″N, 113°56″E) of Henan Agricultural University in 2018. The Yuanyang site is in the center of China, with a warm temperate continental monsoon climate. The previous crop was maize. There were 10 wheat varieties tested, namely, SM159, XN20, XN511, YM11, ZM119, ZM136, ZM158, ZM318, ZM32, and ZM36. The area of the community was 25 × 5 m, and the row spacing was 20 cm. Nitrogen fertilization was applied as ammonium nitrate in the winter at rates of 127.5 kg ha^−1^, and watering once in overwintering period and jointing period respectively.

### Image acquisition

Wheat ear image data were captured during the flowering and filling period (Table 1). Image acquisition was conducted in a Redmi Note 7 mobile phone (Xiaomi, Beijing, China), HUAWEI nova 3i (HUAWEI, Shenzhen, China) and DJI Phantom 3 Pro (DJI, Shenzhen, China). The Redmi Note 7 mobile phone has 48 million + 5 million pixels in the rear cameras, the HUAWEI nova 3i mobile phone has 24 million + 2 million pixels in the rear cameras, and the Phantom 3 Pro has a battery capacity of 23 min for each flight and can take auxiliary hovering pictures. Three devices are high quality with full color. Image acquisition was carried out on both sunny and cloudy days. The image acquisition mode was vertical shooting. The ground resolution was 0.18–1.0. One flight of 3 m altitudes was completed in Xuchang (Table 1), the purpose of which was to verify the portability of the research method on the unmanned aerial vehicle (UAV) platform. One data collection was in Yuanyang in order to verify the applicability of the method in different wheat varieties. On May 13, 2020 in Xuchang, a 12 × 30 cm white board was placed as the ground standard when the image was taken, and one square meter area was selected in the center of the shooting area for wheat ear manual measurement and counting, to verify the applicability of proposed method in field condition.

**Table 1:** Summary of the main image acquisition characteristics of the two experimental sites

Sites	Date	Weather	Plot	Image size	Camera	Image	Focal length (mm)	Resolution (mm)
Xuchang	06/05/2019	Sunny cloudy	12	4000 × 3000	Redmi Note 7	60	5	0.27–0.54
Xuchang	14/05/2019	Sunny cloudy	12	4000 × 3000	Redmi Note 7	60	5	0.27–0.54
Xuchang	14/05/2019	Sunny cloudy	12	4000 × 3000	DJI Phantom 3 Pro	20	4	1.00
Yuanyang	15/05/2019	Sunny cloudy	10	4000 × 3000	Redmi Note 7	490	5	0.27–0.54
Xuchang	16/05/2019	Sunny	12	4000 × 3000	Redmi Note 7	60	5	0.27–0.54
Xuchang	20/05/2019	Sunny	12	4000 × 3000	Redmi Note 7	324	5	0.27–0.54
Xuchang	13/05/2020	Sunny cloudy	12	4608 × 3456	HUAWEI nova 3i	48	4	0.18–0.26

The images collected by the mobile phones were taken by holding mobile phones or holding selfie sticks at an altitude of 1.5–2.2 m. The UAV images were taken at an altitude of 3 m

### Image processing

**Fig. 1:**
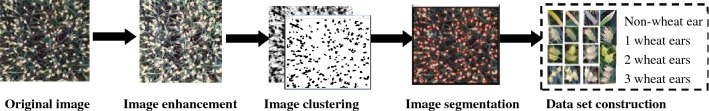
Flow charts of wheat ear image processing. The original image was enhanced using adaptive histogram equalization, then the original image was segmented into four types of data through K-means clustering segmentation: non-wheat ear, one wheat ear, two wheat ears, and three wheat ears

Wheat ear images were processed with image processing technology and were clustered and segmented, following which they were sent to the CNN model for learning and recognition. The algorithm flow chart is shown in Fig. 1.

To accelerate image processing, the original image was reduced by 1400 × 1400 from the center of the acquired image and scaled to 700 × 700. Following enhancement by histogram equalization, the contours of the wheat ears were extracted by K-means clustering segmentation. The segmented images were divided into four categories: non-wheat ear, one wheat ear, two wheat ears, and three wheat ears. Image processing algorithm was developed in Python (3.7, Python Software Foundation) using the OpenCV library (4.2) [23].

### Image denoising and enhancement

Due to the reflection of the wheat leaves under sunlight, the instability of the camera during shooting, and the influence of the natural environment, some noise will appear in the images. In addition, the image may be interfered with by random signals during the transmission process. It was thus necessary to enhance and denoise the wheat ear images.

**Fig. 2:**
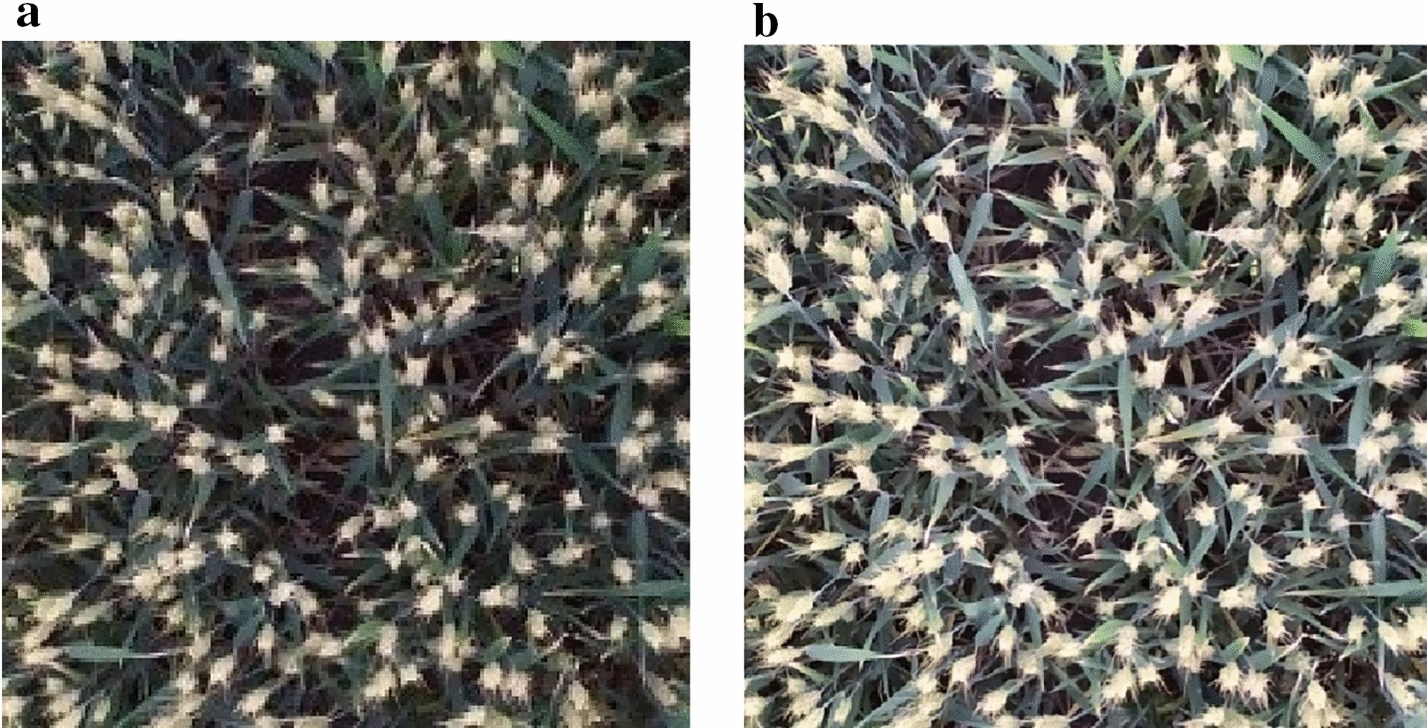
Image denoising and enhancement. **a** Original image **b** enhanced image. The original image was transformed into CIELAB, and the L channel was enhanced using adaptive histogram equalization with a threshold of 2. Enhanced image increases the brightness of the wheat ears to distinguish the background, such as the stem and leaves

The image was transformed into CIELAB [24], and the L channel with a threshold of 2 was used for adaptive histogram equalization to enhance the image (using Python with OpenCV library, the createCLAHE function with parameter clipLimit = 2.0, tileGridSize = (8, 8)), and the size of kernel 3 was used to perform median filtering to remove noise (using Python with OpenCV library, medianBlur function with parameter ksize = 3). Figure 2 shows the original wheat ear image and the enhanced wheat ear image. The wheat ear image is mainly composed of the ear, leaf, stem, and soil, and the ear color characteristics are more obvious when the wheat is in the filling stage. During the filling stage, the wheat ear turns yellow gradually, showing obvious color differences with the leaf and stem, as well as the ground, but the difference between the wheat leaf color and stem color is small (Fig. 2). Enhancing the image increases the brightness of the wheat ears in the image, which makes the contrast between the wheat ear and the background of the stem and leaf more obvious, which is advantageous for the extraction of wheat ears’ features.

### Image segmentation and wheat ear contour extraction

The K-means algorithm is a clustering algorithm based on iterative solution [25–27]. It uses distance as the index of similarity, meaning that the closer the two data points are, the greater the similarity. The traditional method of extracting features by hand is time-consuming and labor-intensive and can easily produce errors in the images of dense wheat ears. In this study, a K-means-based image segmentation algorithm was used for wheat ear segmentation to replace the traditional manual feature extraction of wheat ear color features and thus reduce the error of manual extraction, which was realized in Python Scikit-learn [28] library using the KMeans function.

**Fig. 3:**
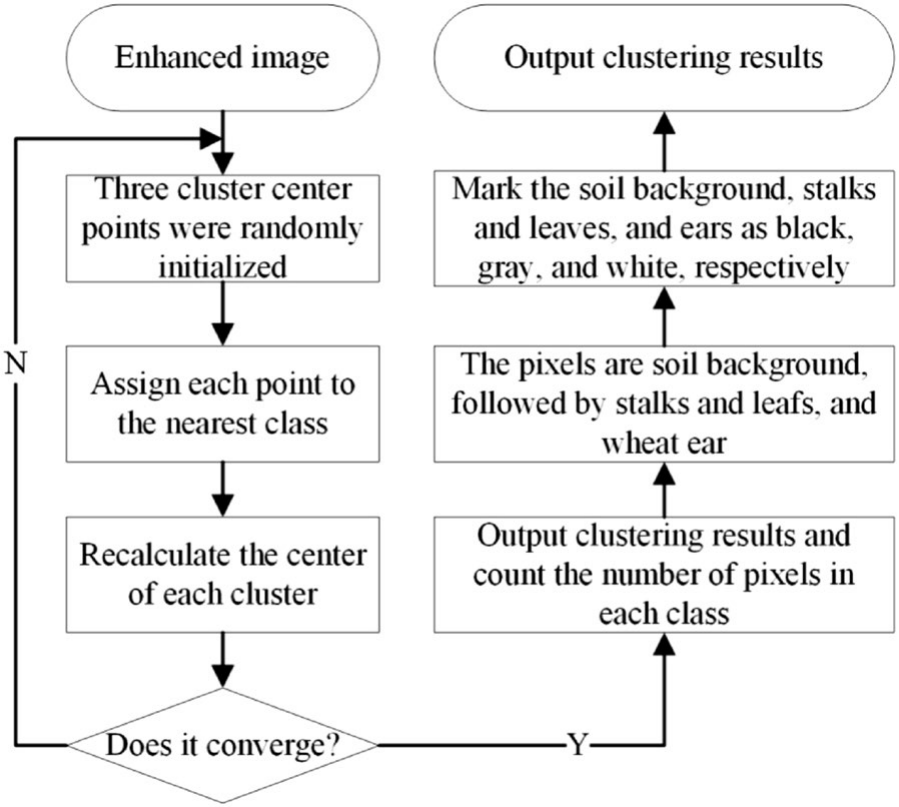
Wheat ear image segmentation algorithm flow. Three segmentation categories are beneficial for the segmentation accuracy: soil background, stem and leaf, and wheat ear, which were realized in Python Scikit-learn library using the KMeans function

After image enhancement, there were obvious differences between the color of the wheat ear and the background color of the stem, leaf, and transition colors. If these are directly clustered into two groups, it will lead to segmentation errors in the color transition area. Therefore, three clustering centers were selected to use K-means clustering to quantify the color of the wheat ear image. After clustering, the wheat ear image will only contain a specified number of categories. The process is as follows: the wheat ear image is clustered, three clustering centers are selected, the clustered wheat ear image is converted into a gray image, and the color of the wheat ear is assigned to black. A flow chart of this process is shown in Fig. 3.

**Fig. 4:**
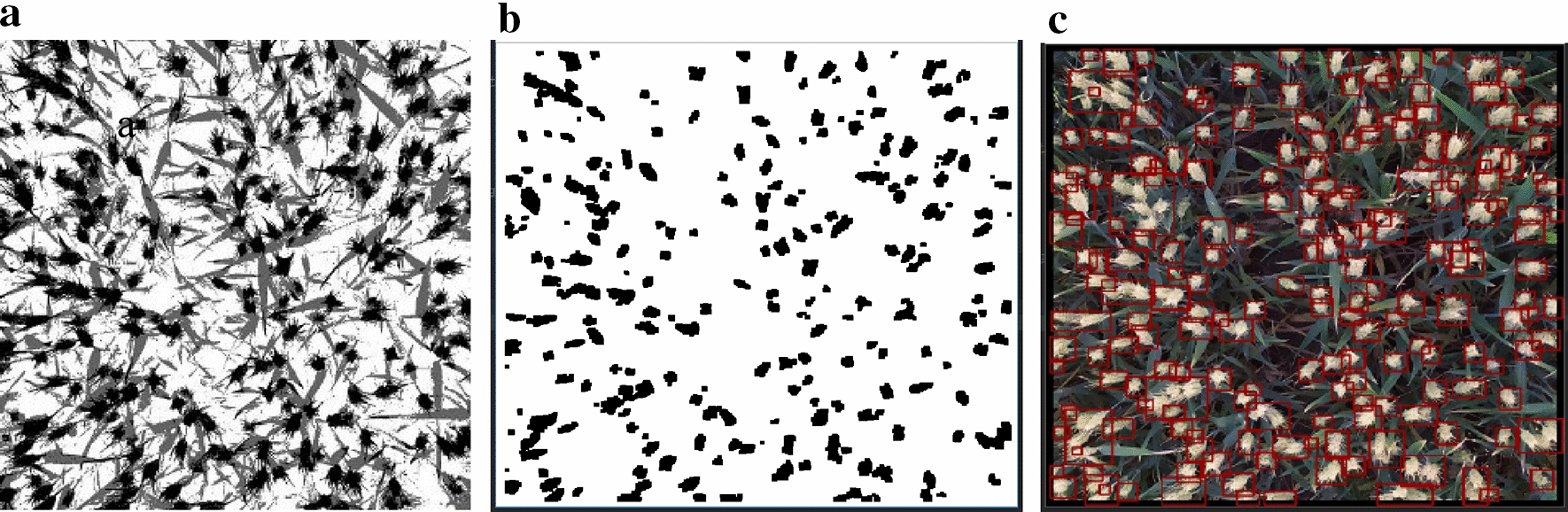
Wheat ear contours feature extraction diagrams. **a** Clustering image, **b** binarization image by removing background noise and the burr around, filling in the holes inside in the wheat ear, **c** masked segmentation image. The image segmentation and contour extraction were developed in the Python OpenCV library using the findContours function, then wheat ears are marked on the original image

According to the color characteristics of the wheat ear after clustering, the image of the wheat ear after clustering is binarized (black for wheat ears, white for the background area, gray for the stalk and leaf). As there is noise in the ear image after segmentation, some of the ears stick to each other. For the binary image, a morphological opening with anchor 6 × 6 was used to remove background noise and the burr around the wheat ear, and then morphological closing with anchor 3 × 3 was used to fill in the holes in the wheat ear, as indicated in Fig. 4a, b. The black area is the contour of the wheat ear after morphological processing.

By comparing the binary image with the original image, the wheat ear image was obtained using the contour feature of the wheat ear and the information of the center of mass, area, perimeter, and boundary frame of each black connected area, which were developed in OpenCV library using the findContours function with parameters contours = 1 and hierarchy = 5. After obtaining the boundary frame of each black connected region, the wheat ears were marked on the original image by a mask, and then the marked wheat ears were divided into small images and saved. A border was added to the original image to prevent the ears near the border from becoming indivisible. A complete wheat ear segmentation map was obtained as shown in Fig. 4c.

### Data set construction

**Fig. 5:**
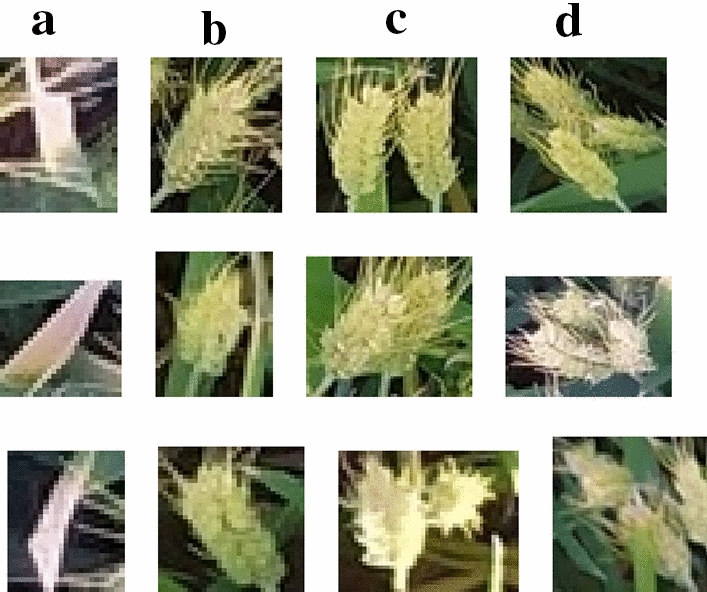
Illustration of the data set images. **a** Non-wheat ear, **b** one wheat ear, **c** two wheat ears, and **d** three wheat ears. The data set images of two and three ears of wheat were mostly the result of adhesion

Seventy of 490 images of Xuchang on May 20, 2019 and 50 of the 324 images of Yuanyang on May 15, 2019 were reserved for testing. The remaining 694 images were segmented into 160,784 small images as a training set and a verification set. Other collected images were used to measure the generalization ability of the method. After batch segmentation, it was found that due to strong light, part of the wheat leaves had strong reflection, resulting in them being mistaken as wheat ears. Second, the wheat ear after segmentation was basically one wheat ear, two wheat ears, or three wheat ears, and more than three wheat ears in one image was rare. Therefore, to reduce complexity in the establishment of the CNN model, the recognition categories were output into four categories: non-wheat ear, one wheat ear, two wheat ears, and three wheat ears. Following the segmentation, the two types of images with more images were non-wheat ears and one wheat ear, whereas the images with more than three wheat ears, particularly three wheat ears, were less. Therefore, to maintain the equilibrium of the data set, four types of wheat ear were selected from the segmentation images. Four categories of labeled image data sets were selected, and the number of non-wheat ear, one wheat ear, two wheat ears, and three wheat ears were 1483, 4246, 1173, and 893, respectively. Some of these results are provided in Fig. 5.

To provide sufficient data for model training, 12,000 augmented images of non-wheat ear, one wheat ear, two wheat ears, and three wheat ears were produced by randomly cutting, flipping, rotating, and adjusting the brightness of the original image [29–31]. The expanded data set was divided into a training and test set, and each class included 11,000 training sets and 1000 test sets.

### CNN model construction and recognition

Deep learning allows the neural network to grasp data features by itself, providing a more abstract high-level representation by combining low-level features to describe the high-level attribute categories or features of the identified objects [32–35]. A large amount of data was available following clustering segmentation, and the segmented image was composed of four types of images: non-wheat ear, one wheat ear, two wheat ears, and three wheat ears. The CNN model was established to train and recognize the four categories of segmented images. Through clustering segmentation, a large number of wheat ear images were obtained and could effectively scale the data without feature engineering. Furthermore, the algorithm exhibited strong adaptability and was easily convertible.

**Fig. 6:**
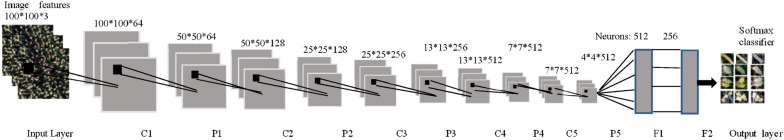
CNN model. *C* convolutional layer, *P* pooling layer, *F* fully connected layer

The CNN model was composed of five convolution layers, five pooling layers, 3 × 3 convolution layer convolution kernels to extract features, and two fully connected layers. The structure is indicated in Fig. 6, the active function is Rectified Linear Unit (ReLU), and the softmax cross entropy loss function is used to quantify the CNN method accurate. Following model training, the images of the test set were segmented after image enhancement, color reversal, and clustering. The trained CNN model was loaded, and the segmented photos were provided to the model for recognition and classification. Then the number of each classification was recorded, finally adding all of the different quantities to obtain the number of ears.

## Statistical analysis

The Xuchang site test data set was divided into three parts: random test, different shooting time, and UAV shooting using SPSS software (25.0, SPSS, Chicago, IBM, USA) (Table 2), and 120 images were used to evaluate the performance. Fifty images of 10 different cultivars in the Yuanyang site data set were used to evaluate the repeatability.

**Table 2:** Summary of the manual counting of ears of winter wheat at the Xuchang and Yuanyang experimental sites

Sites	Data set	Samples size	Min	Mean	Max	Range	SD	CV (%)
Xuchang	*Test*	60	166	250	357	191	43	17
	May 6	10	173	239	309	136	47	20
	May 14	10	137	195	232	95	30	15
	May 16	10	251	291	326	75	26	9
	May 20	10	194	233	275	81	29	13
	*All date*	40	137	240	326	189	48	20
	*UAV*	20	117	176	255	138	43	24
Yuanyang	SM159	5	274	315	367	93	33	11
	XN20	5	316	346	387	71	27	8
	XN511	5	265	322	362	97	39	12
	YM11	5	289	318	360	71	27	8
	ZM119	5	219	254	271	52	23	9
	ZM136	5	276	301	320	44	16	5
	ZM158	5	220	250	285	65	27	11
	ZM318	5	202	280	310	108	45	16
	ZM32	5	265	338	378	113	44	13
	ZM36	5	232	270	303	71	27	10
	*All cultivars*	50	202	300	387	185	44	15
	*All*	170	117	253	387	270	58	23

The cultivar image of Yuanyang was acquired on May 15, 2019, whereas the UAV image of Xuchang was acquired on May 14, 2019

*SD* standard deviation, *CV* coefficient of variation

To evaluate the classification performance of the CNN model, the precision (*P*), recall (*R*), macro F1-score (*F*_1,*ma*_), and micro F1-score (*F*_1,*mi*_) were calculated to evaluate the performance of multi-label classification model [36, 37], which are defined as follows:

1 $${P}_{i}= \frac{{TP}_{i}}{{TP}_{i}+{FP}_{i}}$$

2 $${R}_{i}= \frac{{TP}_{i}}{{TP}_{i}+{FN}_{i}}$$

3 $${F}_{1,ma}=\frac{2}{n}\sum_{i=1}^{n}\frac{{P}_{i}\times {R}_{i}}{{P}_{i}+{R}_{i}}$$

4 $${F}_{1,mi}= 2\times \frac{{P}_{mi}\times {R}_{mi}}{{P}_{mi}+{R}_{mi}}$$

$${P}_{mi}= \frac{ \sum_{i=1}^{n}T{P}_{i}}{\sum_{i=1}^{n}{TP}_{i}+\sum_{i=1}^{n}{FP}_{i}}$$

$${R}_{mi}= \frac{ \sum_{i=1}^{n}T{P}_{i}}{\sum_{i=1}^{n}{TP}_{i}+\sum_{i=1}^{n}{FN}_{i}}$$

where *TP*_*i*_*, is* true positive, which denotes the number of images correctly classified as change type *i*; *FP*_*i*_is false positive, which denotes the number of images incorrectly classified as change type *i*; and *FN*_*i*_is the false negative for class *i*, which denotes the number of images of type *i* that are incorrectly classified as other types. *P*_*i*_ and *R*_*i*_ are respectively precision and recall for class *i*, *n* is the number of classes (this study, *n* = 4), and *P*_*mi*_ and *R*_*mi*_ are respectively precision and recall for Micro-F1.

In addition, *R*^2^ and RMSE, the relative root means square error (RRMSE) [38], and bias were used to quantify the counting performance of the model:

5 $${R}^{2}=1-\frac{{\sum }_{i=1}^{n}{\left({m}_{i}-{c}_{i}\right)}^{2}}{{\sum }_{i=1}^{n}{\left({m}_{i}-\stackrel{-}{m}\right)}^{2}}$$

6 $${\text{RMSE}}=\sqrt{\frac{1}{n}\sum_{i=1}^{n}{\left({m}_{i}-{c}_{i}\right)}^{2}}$$

7 $${\text{RRMSE}}=\frac{RMSE}{\stackrel{-}{m}} \times 100\%$$

8 $${\text{Bias}}=\frac{1}{n}\sum_{i=1}^{n}\left({m}_{i}-{c}_{i}\right)$$

where n is the number of images, and ${{\varvec{m}}}_{{\varvec{i}}}$ and ${{\varvec{c}}}_{{\varvec{i}}}$ are the manual annotation and identifying counts for image i, respectively, and $\stackrel{-}{{\varvec{m}}}$ is the average of the manual annotation counts.

## Results

The CNN framework was trained and tested in PyCharm (2019.3, PyCharm, Prague, JetBrains, Czech) using the TensorFlow framework (TensorFlow1.15, Google, California, USA) on a Windows 10 PC Intel Core i7 processor (3.6 GHz) with 16 GB RAM. In this paper, a 1400 × 1400 image was cut from the original image from the center position and then scaled to 700 × 700. After segmentation, the four categories images were uniformly scaled to 100 × 100. On this basis, the performance evaluation of the CNN machine learning method could be compared to the manual annotation and counting of the image.

### Model accuracy evaluation

**Fig. 7:**
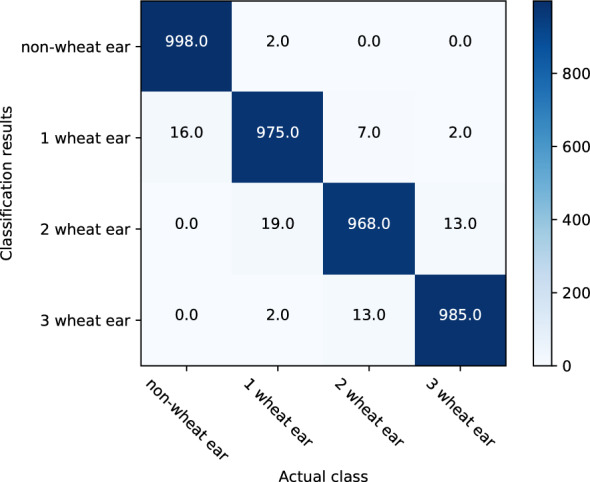
The multiclass confusion matrix of the different classification results using the test data. The method achieved good results in the classification of each category

To assess the classification results, after 8000 epochs of training, we adopted indices of macro F1-score and micro F1-score calculated on a multiclass confusion matrix. The classification results obtained by the methods are shown in Fig. 7 and Table 3. Figure 7 lists the confusion matrix in detail, which calculates the statistics of the classified image number by comparing the actual label in the test data with the predicted types and indicates whether the model is confounding the different classes. In Table 3, the precision, recall, and F1-score of the four classes are calculated on the basis of the confusion matrix, which embodies the classification accuracy of each class. From the table, we notice that the macro F1-score and micro F1-score both achieved 98.47%. From this result, we can infer that the recognition accuracies of non-wheat ear, one wheat ear, two wheat ears, and three wheat ears were 99.8, 97.5, 98.07, and 98.5%, respectively.

**Table 3:** Quantitative comparison of the classification accuracy for different classes using the test data

Class	Precision (%)	Recall (%)	F1-score (%)	Macro F1-score (%)	Micro F1-score (%)
Non-wheat ear	99.80	98.42	99.11	98.47	98.47
1 wheat ear	97.50	97.70	97.60		
2 wheat ears	98.07	97.98	98.03		
3 wheat ears	98.50	99.80	99.14		

The recognition accuracy of non-wheat ear, one wheat ear, two wheat ears, and three wheat ears all have higher precision

### Evaluation of performance of wheat ear images

**Fig. 8:**
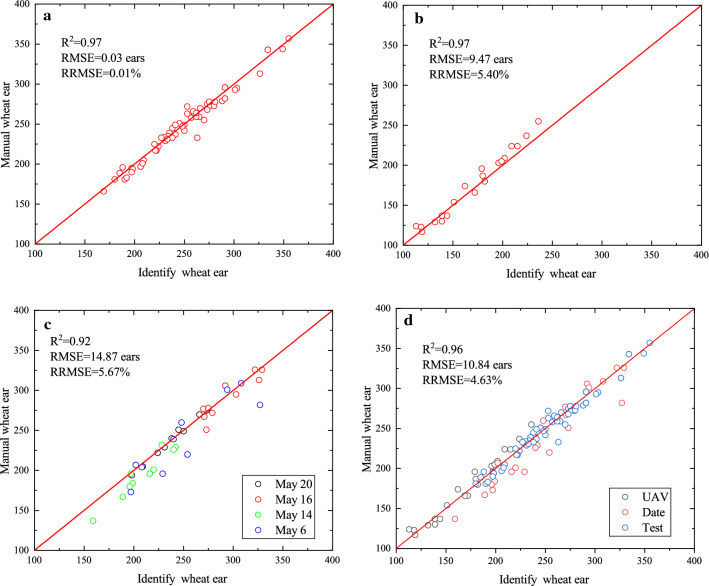
Comparison between the wheat ears identified using the model with the corresponding manual values by visually identifying the ears in the images. Data for the three experimental data sets are well identified. **a** Test images, **b** UAV images, **c** different date images, and **d** all test images in Xuchang

Test images were preprocessed and clustered, and then each image was segmented, saved, and sent to the CNN model for recognition and counting to test the generalization ability of the model. The comparison between the detected ears of wheat images and the manual counting results is shown in Fig. 8.

The performances evaluated over the test data sets showed only a slight degradation in comparison with the test and different datasets, providing some confidence on the robustness of the K-means-CNN method (Fig. 8 and Table 4). The model-based identification of the wheat ears was in good agreement with the manual identification (Fig. 8a and Table 4). The result demonstrated that the high *R*^2^ = 0.96 of the K-means-CNN counting was highly correlated with manual counting and demonstrated low data dispersion (Table 4).

**Table 4:** Relationships between the identified and manual wheat ear counting for the three data sets

Sites	Dataset	Samples size	Slope	Intercept	RMSE (ears)	R^2^	RRMSE (%)	Bias (ears)
Xuchang	*Test*	60	0.97	8.53	0.03	0.97	0.01	1.40
	May 6	10	0.88	41.25	22.54	0.82	9.43	6.90
	May 14	10	0.82	47.97	16.10	0.93	8.26	11.60
	May 16	10	0.85	46.10	10.30	0.84	3.54	2.90
	May 20	10	0.91	21.79	3.24	0.99	1.39	0.10
	*All dates*	40	0.88	34.68	14.87	0.92	6.21	7.10
	*UAV*	20	0.87	18.52	9.47	0.97	5.40	-5.00
	*All*	120	0.97	9.05	10.84	0.96	4.63	2.24

The K-means-CNN counting was highly correlated with manual counting

However, performances of identify degrade for the different dates of grain filling stage (Table 4). The bias between the identified and the manual ear values ranged from 0.1 ears (May 20) to 11.60 (May 14) ears for Xuchang (Table 4). The poorer performances observed on May 6 (*R*^2^ = 0.82, RMSE = 22.54) may be attributed to the early stage when the wheat ear is not yet mature. In these conditions, the contrast between the wheat ears with the stems and leaves is poor, whereas the characteristics are more obvious and easily identifiable in the later stages, and thus the best performance was observed on May 20 (*R*^2^ = 0.99, RMSE = 3.24, Table 3). The results suggested that the images should be taken at the later grain-filling stage around May 20. Our results are in good agreement with those of earlier studies [11].

**Fig. 9:**
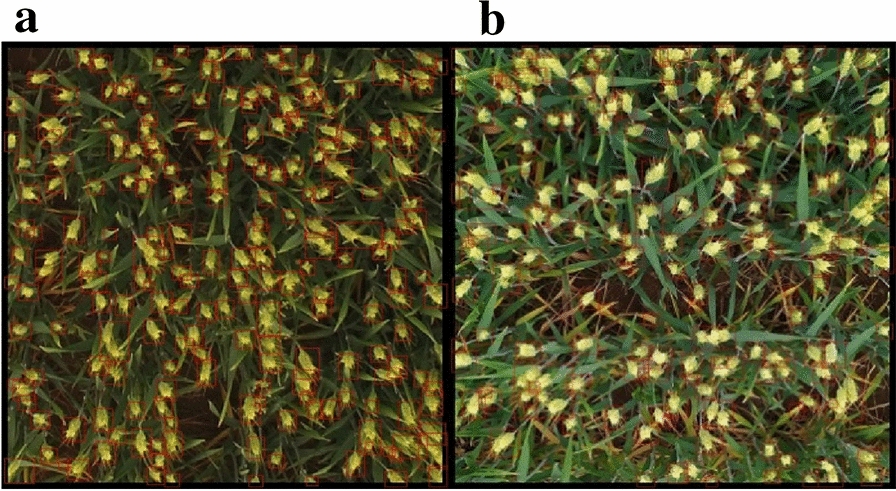
Image segmentation and wheat ear recognition. **a** Segmentation and recognition of the UAV image, **b** segmentation and recognition of the mobile phone image. The images collected by the UAV and hand-held device all achieved high recognition accuracy

To further evaluate the robustness of the proposed method, 20 UAV images not involved in training were used for verification. The relationship between the K-means-CNN model and manual ear counting was positive and strong, with an *R*^2^ of 0.97 and an RMSE of 9.47 ears. This result showed that the images collected by the UAVs and hand-held devices all achieved high recognition accuracy using the proposed method (Fig. 9). In addition, the UAV data set bias values were –5.00, indicating a slight overestimation of the number of ears.

### Repeatability across different cultivars

**Fig. 10:**
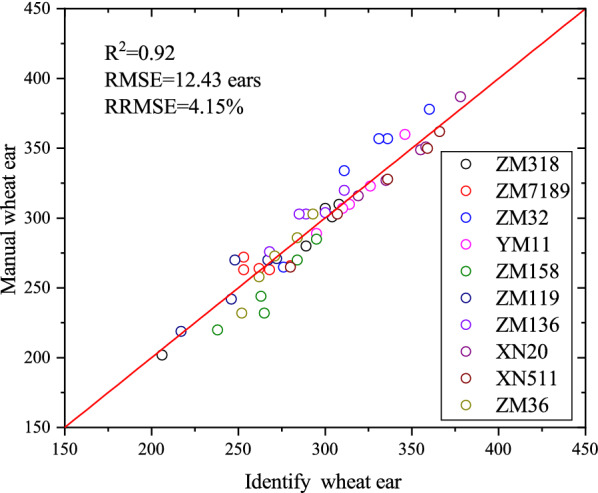
Comparison of wheat ear identification from the images of 10 different cultivars extracted from the same subsample

Fifty subsamples with 10 different cultivar extracts of the subsample were selected in the Yuanyang site to evaluate the repeatability of the estimation when the images were taken under slightly different cultivation conditions. High consistency between the 10 cultivars was observed (Fig. 10), with the residuals showing a standard deviation of about 12.43 ears.

The performance of the algorithm was further tested using the 50 images. Manual counting was used as the validation data, as before. Table 5 provides the statistical summary results obtained for the Yuanyang plots. The results showed a decrease by up to 0.04 in *R*^2^ while maintaining a similar correlation, and the bias between the identified and the manual ear values ranged from − 15.40 ears (ZM32) to 18.80 (ZM158) ears for Yuanyang (Table 4). The *R*^2^ value remained close to the Xuchang values for all but the ZM119 and ZM136 images, where the correlation values shifted slightly from the original values. The best performance was observed in XN511 (*R*^2^ = 0.99, RMSE = 8.97, Table 5), and the lowest was observed in ZM119 (*R*^2^ = 0.81, RMSE = 10.14, Table 5). This suggested that the genotypes of the different cultivars will slightly affect the identification results. These results suggest that more genotype images are needed to contribute to model training to achieve higher accuracy.

**Table 5:** Relationships between the identified and manual counting of 10 cultivars

Sites	Data set	Samples size	Slope	Intercept	RMSE (ears)	*R*^2^	RRMSE (%)	Bias (ears)
Yuanyang	SM159	5	0.61	127.37	12.47	0.98	3.96	3.60
	XN20	5	0.81	67.88	6.91	0.96	2.00	3.00
	XN511	5	0.92	32.27	8.97	0.99	2.79	8.00
	YM11	5	0.71	92.03	7.29	0.98	2.30	0.40
	ZM119	5	0.84	36.91	10.14	0.81	3.99	− 4.40
	ZM136	5	0.96	0.33	11.67	0.89	3.87	− 10.60
	ZM158	5	0.78	74.03	20.35	0.92	8.13	18.80
	ZM318	5	0.94	18.50	5.64	0.98	1.91	1.40
	ZM32	5	0.70	86.44	20.45	0.95	6.05	− 15.40
	ZM36	5	0.60	110.48	10.24	0.97	3.79	2.00
	All cultivars	50	0.85	45.09	12.43	0.92	4.15	0.68

ZM119, ZM136, and ZM32 overestimated the number ears

### Evaluation of performances in field condition

**Fig. 11:**
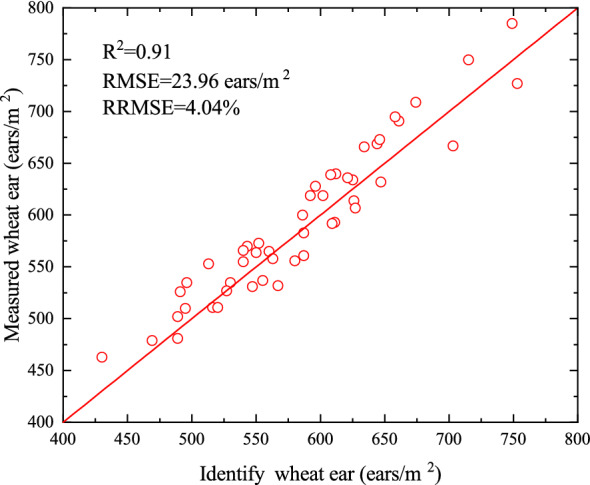
Comparison between the wheat ears identified using the model with the corresponding values by manual counting in field condition. 48 samples are highly correlated with measurement counts in the field condition

Forty-eight subsamples of wheat ear images with ground standard were selected in the Xuchang site to evaluate the accuracy and practicality of the proposed method. It can be concluded that 48 samples are highly correlated with measurement counts in the field condition (Fig. 11).

The performance of the method was further tested using 48 subsamples. One Square meter area was selected in the center of the image area for manual counting in the field condition, which was used as the test data. Figure 11 shows the results obtained, with the residuals showing a standard deviation of about 23.96 ears/m^2^, the relationship between the method and measurement ear counting was positive and strong, with an *R*^2^ of 0.91 and RRMSE of 4.04%. The results showed a decrease in *R*^2^, indicating a slight reduction the identification results in field condition. The reason may be related to the small number of wheat ears hidden under the stems and leaves during field counting.

## Discussion

The results showed that the number of wheat ears identified by K-means and CNN was consistent with the manual ear counting results (Fig. 8 and Table 4). The difference between the two methods (Fig. 8) indicated that the accuracy is poor in the earlier grain-filling stage. The results of Alkhudaydi et al. [39] also suggested that this model performed well during the grain-filling stage. These results confirm that better-quality images can be obtained from the later grain-filling stage.

Our method is based on target localization. Adding a later stage would probably have led to a marginal improvement, as the ears in the grain-filling stage are a relatively homogeneous yellow color. Furthermore, the images were grouped into three groups to avoid the discarded region where the contrast between the ear and background is not great enough in the K-means segmentation. The identification error caused by the adhesion of the wheat ear and background proposed by Fernandez-Gallego et al. [11] was effectively reduced. In addition, the wheat ear images were divided into non-wheat ear, one wheat ear, two wheat ears, and three wheat ears, which could effectively reduce the identification inaccuracy caused by the adhesion of multiple wheat ears, which has been a significant issue in traditional image processing methods [10].

Overall, the proposed K-means and CNN algorithm showed suitable performance in identifying wheat ears at early or later growth stages in all datasets (*R*^2^ = 0.96, RMSE = 10.84 ears, Table 3), and similar outcomes were presented by Zhou [40]. The result using K-means to segment the wheat ear features accurately and train the machine learning model not only improved the model training efficiency but also improved the recognition accuracy. This method was used to classify the wheat ear instead of using target recognition to reduce the complexity of the algorithm, and together with the CNN model, could effectively and accurately identify and count the wheat ears.

Our work is useful for the development of a low-cost, rapid, and easy-to-implement method to identify wheat ears. We used images collected by the UAV platform to verify the training model of the mobile phone photo collection, which also achieved good results. However, determining the actual area represented in the photos still needs to be resolved. Current research mainly uses measures such as placing a reference substance as a ground standard [22] or fixing the shooting height [18], which reduces the practicability of the method. In the future, augmented reality (AR) technology could be used to solve this problem, which is one of our research aims.

It should be noted that different cultivars had a slight influence on the identification results. Although the training data of the CNN model were constructed on May 14, 2019 and May 20, 2019, and thus the sample size of the training data set was not large, the model still achieved good recognition of the images collected on the other dates. In our opinion, the best shooting date is at the late stage of grouting, when the wheat ears turn yellow and the stems and leaves are still green. In addition, we believe that the use of mobile devices to shoot images at the height of 1.5–2.2 m in sunny cloudy is a better way of shooting, as it matches the height of the person and facilitates the practical application of this method.

## Conclusion

In this study, wheat ear images were collected using hand-held equipment, which is fast and convenient. Through K-means clustering segmentation, complete wheat ear images were automatically segmented, and automatic feature extraction of the wheat ear images was realized. The code can be found at https://github.com/xuxin468/earcouting.

The segmented images were divided into four types, and the CNN model was established to realize the recognition and counting of the wheat ear images. The correlation coefficient *R*^2^ was 0.96. The recognition accuracies of the non-wheat ear, one wheat ear, two wheat ears, and three wheat ears were 99.8, 97.5, 98.07, and 98.5%, respectively. The results showed that the recognition accuracy of the CNN model could be improved by using image processing technology to accurately locate and segment wheat ears before training and recognizing, thus meeting the requirements of field-based wheat ear counting.

The present study has several improvements over previous studies: (1) K-means clustering was used to automatically and accurately segment the wheat ear, thus reducing the traditional workload of manual labeling and the associated human errors. (2) The wheat ear adhesion problem was resolved by creating four types of labeled datasets, including the non-wheat ear, single wheat ear, two wheat ears, and three wheat ears, which transforms the task of wheat ear recognition into the task of wheat ear image classification. (3) K-means was used to segment the wheat ear features accurately, and as a result, the efficiency and accuracy of the machine learning model was significantly improved.

The wheat ear recognition model based on CNN demonstrates strong generalization ability and robustness and can be applied to UAV platform as well. This paper combined automatic image processing and CNN methods, which is of great technical value for the recognition and counting of wheat ears in the field.

Our aim was to help reduce the cost of image acquisition and improve the application scope of this method. This method can be used to estimate wheat ear numbers and improve the efficiency of wheat yield estimation. At the same time, it can also provide breeders with a fast and automated high-throughput wheat ear counting system to improve breeding efficiency. Although this method is applied to the segmentation and counting of wheat ears, it can also be applied to the segmentation and counting of other plants. In future work, our aim is to use AR measurement technology, which can provide a ground standard for the images.

## Data Availability

The data sets used and/or analyzed during the current study available from the corresponding author on reasonable request.

## References

[1] Nerson H (1980). Effects of population density and number of ears on wheat yield and its components. Field Crop Res.

[2] Zhang HP, Turner NC, Poole ML, Asseng S (2007). High ear number is key to achieving high wheat yields in the high-rainfall zone of south-western Australia. Aust J Agric Res.

[3] Ferrante A, Cartelle J, Savin R, Slafer GA (2017). Yield determination, interplay between major components and yield stability in a traditional and a contemporary wheat across a wide range of environments. Field Crop Res.

[4] Li L, Zhang Q, Huang DF (2014). A review of imaging techniques for plant phenotyping. Sensors.

[5] Grift TE, Zhao W, Momin MA, Zhang Y, Bohn MO (2017). Semi-automated, machine vision based maize kernel counting on the ear. Biosyst Eng.

[6] Mochida K, Koda S, Inoue K, Hirayama T, Tanaka S, Nishii R, Melgani F (2019). Computer vision-based phenotyping for improvement of plant productivity: a machine learning perspective. GigaScience.

[7] Knecht AC, Campbell MT, Caprez A, Swanson DR, Walia H (2016). Image Harvest: an open-source platform for high-throughput plant image processing and analysis. J Exp Bot.

[8] Pearline SA, Kumar VS, Harini S (2019). A study on plant recognition using conventional image processing and deep learning approaches. J Intell Fuzzy Syst.

[9] Perez-Rodriguez F, Gomez-Garcia E (2019). Codelplant: Regression-based processing of RGB images for colour models in plant image segmentation. Comput Electron Agric.

[10] Cointault F, Guerin D, Guillemin JP, Chopinet B (2008). In-field Triticum aestivum ear counting using colour-texture image analysis. N Z J Crop Hortic.

[11] Fernandez-Gallego JA, Kefauver SC, Gutierrez NA, Nieto-Taladriz MT, Araus JL (2018). Wheat ear counting in-field conditions: high throughput and low-cost approach using RGB images. Plant Methods.

[12] Fernandez-Gallego JA, Buchaillot ML, Gracia-Romero A, Vatter T, Diaz OV, Gutierrez NA, Nieto-Taladriz MT, Kerfal S, Serret MD, Araus JL, Kefauver SC (2019). Cereal crop ear counting in field conditions using zenithal RGB images. J Vis Exp.

[13] LeCun Y, Bengio Y, Hinton G (2015). Deep learning. Nature.

[14] Jo JW, Hye LM, Hong-Ro L, Suk CY, Baek JH, Kim KH, Lee CW (2019). LeafNet: plants segmentation using CNN. J Korea Soc Ind Inform Syst.

[15] Zhu YX, Sun WM, Cao XY, Wang CY, Wu DY, Yang Y, Ye N (2019). TA-CNN: two-way attention models in deep convolutional neural network for plant recognition. Neurocomputing.

[16] Zhu YJ, Cao ZG, Lu H, Li YN, Xiao Y (2016). In-field automatic observation of wheat heading stage using computer vision. Biosyst Eng.

[17] Li QY, Cai JH, Berger B, Okamoto M, Miklavcic SJ (2017). Detecting spikes of wheat plants using neural networks with Laws texture energy. Plant Methods.

[18] Hasan MM, Chopin JP, Laga H, Miklavcic SJ (2018). Detection and analysis of wheat spikes using convolutional neural networks. Plant Methods.

[19] Madec S, Jin XL, Lu H, De Solan B, Liu SY, Duyme F, Heritier E, Baret F (2019). Ear density estimation from high resolution RGB imagery using deep learning technique. Agric Forest Meteorol.

[20] Hamuda E, Glavin M, Jones E (2016). A survey of image processing techniques for plant extraction and segmentation in the field. Comput Electron Agric.

[21] Wang ZB, Li HL, Zhu Y, Xu TF (2017). Review of plant identification based on image processing. Arch Comput Method E.

[22] Sadeghi-Tehran P, Virlet N, Ampe EM, Reyns P, Hawkesford MJ (2019). DeepCount: in-field automatic quantification of wheat spikes using simple linear iterative clustering and deep convolutional neural networks. Front Plant Sci.

[23] Bradski G (2000). The OpenCV library. Dr Dobbs J Softw Tools.

[24] CIE. 015:2018 Colorimetry. 4th ed. Vienna: The International Commission on Illumination; 2018.

[25] MacQueen J (1967). Some Methods for Classification and Analysis of MultiVariate Observations. Proc Fifth Berkeley Symp Math Stat Probab.

[26] Erisoglu M, Calis N, Sakallioglu S (2011). A new algorithm for initial cluster centers in k-means algorithm. Pattern Recogn Lett.

[27] Reza MN, Na IS, Baek SW, Lee KH (2019). Rice yield estimation based on K-means clustering with graph-cut segmentation using low-altitude UAV images. Biosyst Eng.

[28] Pedregosa F, Varoquaux G, Gramfort A, Michel V, Thirion B, Grisel O, Blondel M, Prettenhofer P, Weiss R, Dubourg V, Vanderplas J, Passos A, Cournapeau D, Brucher M, Perrot M, Duchesnay E (2011). Scikit-learn: machine learning in python. J Mach Learn Res.

[29] Wang X, Wang K, Lian S (2020). A survey on face data augmentation for the training of deep neural networks. Neural Comput Appl..

[30] Tang C (2020). PLANET: improved convolutional neural networks with image enhancement for image classification. Math Prob Eng..

[31] Fu Y, Li X, Ye Y (2020). A multi-task learning model with adversarial data augmentation for classification of fine-grained images. Neurocomputing.

[32] Deng L, Yu D (2014). Deep learning: methods and applications. Found Trends Signal Process..

[33] Kamilaris A, Prenafeta-Boldu FX (2018). A review of the use of convolutional neural networks in agriculture. J Agric Sci.

[34] Krizhevsky A, Sutskever I, Hinton GE (2017). ImageNet classification with deep convolutional neural networks. Commun Acm.

[35] Ma L, Liu Y, Zhang XL, Ye YX, Yin GF, Johnson BA (2019). Deep learning in remote sensing applications: a meta-analysis and review. Isprs J Photogramm Remote Sens.

[36] Yang Y (1999). An evaluation of statistical approaches to text categorization. Inf Retr.

[37] Menzies T, Dekhtyar A, Distefano J, Greenwald J (2007). Problems with precision: a response to “comments on ‘data mining static code attributes to learn defect predictors’”. IEEE T Softw Eng.

[38] Despotovic M, Nedic V, Despotovic D, Cvetanovic S (2016). Evaluation of empirical models for predicting monthly mean horizontal diffuse solar radiation. Renew Sustain Energy Rev.

[39] Alkhudaydi T, Reynolds D, Griffiths S, Zhou J, Iglesia B (2019). An exploration of deep-learning based phenotypic analysis to detect spike regions in field conditions for UK Bread Wheat. Plant Phenom..

[40] Zhou CQ, Liang D, Yang XD, Yang H, Yue JB, Yang GJ (2018). Wheat ears counting in field conditions based on multi-feature optimization and TWSVM. Front Plant Sci.

